# Toward the Development of a Sustainable Scientific Research Culture in Azerbaijan (2011–2015)

**DOI:** 10.3389/fpubh.2016.00144

**Published:** 2016-07-04

**Authors:** Saida Aliyeva, Peter Flanagan, April Johnson, Lisa Strelow

**Affiliations:** ^1^Civilian Research Development Foundation, Baku, Azerbaijan; ^2^Signature Science, LLC, Baku, Azerbaijan; ^3^Bechtel National. Inc., Baku, Azerbaijan

**Keywords:** defense threat reduction agency, Cooperative Biological Engagement Program, Azerbaijan, research, surveillance

## Abstract

This review especially describes the dangerous pathogens research program in Azerbaijan (AJ) funded by the US Defense Threat Reduction Agency under the Cooperative Biological Engagement Program (CBEP) from 2011 through 2015. The objectives of the CBEP are to prevent the proliferation of biological weapons; to consolidate and secure collections of dangerous pathogens in central repositories; to strengthen biosafety and biosecurity of laboratory facilities; and to improve partner nations’ ability to detect, diagnose, report, and respond to outbreaks of disease caused by especially dangerous pathogens. One of the missions of the CBEP is therefore to increase the research skills and proficiency of partner country scientists. The program aims to fulfill this mission by sponsoring scientific research projects that exercise the modern diagnostic techniques available in the CBEP-engaged laboratories and the enhanced disease surveillance/control programs. To strengthen the local scientists’ ability to develop research ideas, write grant proposals, and conduct research independently, in-country CBEP integrating contractor personnel have mentored scientists across AJ and conducted workshops to address technical gaps. As a result of CBEP engagement, seven research projects developed and led by AJ scientists have been funded, and five projects are currently in various stages of implementation. The Defense Threat Reduction Agency has also sponsored AJ scientist participation at international scientific conferences to introduce and integrate them into the global scientific community. The efforts summarized in this review represent the first steps in an ongoing process that will ultimately provide AJ scientists with the skills and resources to plan and implement research projects of local and regional relevance.

## Introduction

The Defense Threat Reduction Agency (DTRA)’s Cooperative Biological Engagement Program (CBEP) is a US Department of Defense program that is part of the larger Cooperative Threat Reduction Program aimed at reducing chemical, biological, and nuclear threats. In former Soviet Union countries, the CBEP has demolished bioweapon production facilities, consolidated collections of dangerous pathogens, and supported peaceful research activities that employ knowledgeable personnel (1). The CBEP emphasizes scientific engagement as a means to improve biosafety, biosecurity, and disease surveillance capabilities through research, training, technology transfer, infrastructure improvement, and sustainment activities with the goal of reducing the risks associated with infectious diseases and disease outbreaks caused by bioterrorism or endemic threats to public and animal health. As part of the Cooperative Threat Reduction Program, the CBEP works with Azerbaijan (AJ) and other partner countries to establish modern disease surveillance systems and a corps of scientists to study and develop countermeasures against endemic especially dangerous pathogens (EDPs).

The US Government, in cooperation with international partner institutes, has adopted a strategy that is designed to identify and promote CBEP research activities that are of mutual interest to the US Government and host countries. The goals of this partnership are to (1) support efforts to build biosurveillance and biosafety capabilities, (2) engage partner country scientists in hypothesis-driven research, (3) promote One Health initiatives, and (4) foster an international culture of responsible and ethical conduct in biological research. To meet these goals, the CBEP has upgraded laboratory facilities and implemented a comprehensive training program to ensure that AJ scientists are exposed to best practices and are equipped to carry out their disease surveillance responsibilities into the future. The CBEP has created and delivered training modules to enhance the diagnostic and epidemiological capabilities of AJ’s scientific and technical staff and to promote biosafety and biosecurity (BS&S). The CBEP has also employed a training-of-trainers approach to foster long-term sustainability of the program.

The scientific community in AJ – composed of academic research institutes, universities, non-governmental organizations, and other international research funders (such as World Bank) – has a long history and is too complex to describe within the scope of this review. Therefore, only CBEP’s role in AJ is discussed herein.

## Science and CBEP in Azerbaijan

**Figure 1:**
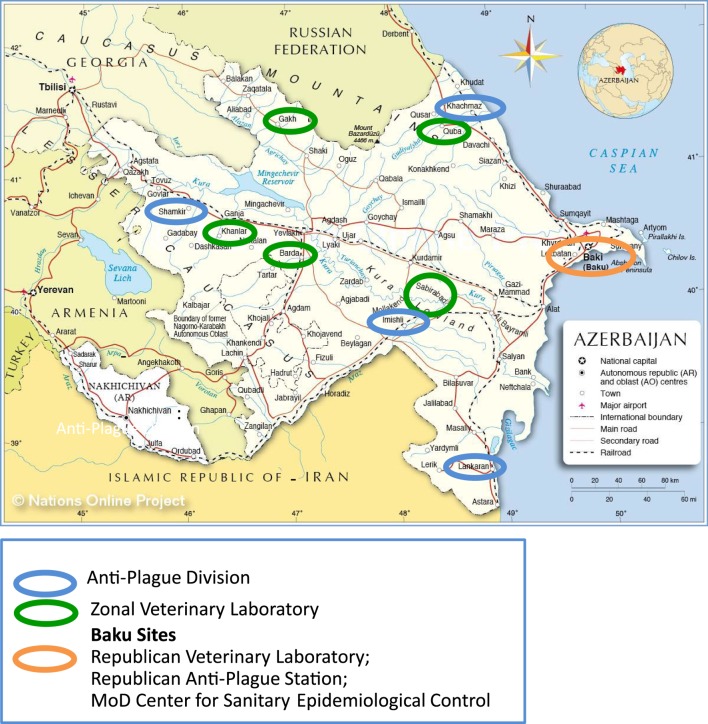
**CBEP-engaged laboratories in Azerbaijan**. Green circles represent the Zonal Veterinary Laboratories; red circles represent the anti-plague divisions; the orange circle represents the Republican level Laboratories including one laboratory each from MoH (Republican Anti-Plague Station), one SVCS (Republican Veterinary Laboratory), and one MoD (Center for Sanitary Epidemiological Control).

The CBEP was first implemented in AJ in 2007. Initially, the program provided funding to renovate or build 12 BSL-2 laboratories belonging to the Azerbaijan Ministry of Health, State Veterinary Control Service (SVCS) under the Ministry of Agriculture, and Ministry of Defense, so as to provide the physical infrastructure for the implementation of BSL-2 diagnostics and research (Figure 1). In 2011, Bechtel National, Inc. (BNI) became the CBEP integrating contractor in AJ. This review therefore describes the CBEP especially dangerous pathogens research program in AJ from 2011 through 2015 only.

At the national level, the Republican Anti-Plague Station (RAPS) and Republican Veterinary Laboratory (RVL) in Baku serve as the top-tier diagnostic institutions in AJ. The RAPS and RVL conduct confirmatory testing for EDPs and other diseases affecting humans and animals, respectively. Outside of Baku, the human threat agent detection and response system is represented by four regional, CBEP-enhanced Anti-Plague Division (APD) laboratories in Imishli, Khachmaz, Lankaran, and Shamkir. Similarly, the veterinary laboratory system outside of Baku is represented by the Zonal Veterinary Laboratories (ZVLs); the five CBEP-enhanced ZVLs are located in Barda, Gakh, Goygol, Guba, and Sabirabad. Each of the Baku and regional CBEP-enhanced laboratories is responsible for diagnosing EDPs and other infections in their home regions using modern molecular, immunological, and bacteriological methods. All of the CBEP facilities enter the laboratory test results for suspect EDP cases into the Electronic Integrated Disease Surveillance System (EIDSS), an electronic reporting system introduced by CBEP and implemented throughout the country.

Beyond the CBEP-renovated laboratories, AJ scientists from other facilities are also involved in CBEP research and training programs. These institutes include the Azerbaijan Veterinary Scientific Research Institute (AVSRI), which is responsible for veterinary biologic preparations and veterinary research; Azerbaijan State Agrarian University (ASAU), which offers veterinary education; Regional Veterinary Offices, which monitor animal health and report to the SVCS; and the Centers for Hygiene and Epidemiology, which monitor human diseases in the country and report to the MoH. Including these institutes in CBEP activities helps to encourage collaboration with the CBEP-engaged laboratories, with the goal of enhancing the national disease surveillance system.

In addition to laboratory renovations and research, CBEP administers an extensive training program to ministry-level and regional facility staff involved with EIDSS. In addition to EIDSS training, CBEP-training subjects include clinical recognition of infections caused by EDPs in humans and animals; epidemiology and veterinary epidemiology; BS&S; and PCR, bacteriology, and serology laboratory diagnostics. A description and evaluation of training conducted by CBEP in 2014 and 2015 was recently published by Johnson et al. (2).

## CBEP Research in Azerbaijan

Cooperative Biological Engagement Program research focuses on the specific pathogens listed in Table 1. Research activity in AJ has focused on EDPs of particular importance to AJ and the Trans-Caucasus region, including *Brucella, Bacillus anthracis, Yersinia pestis, Francisella tularensis*, avian influenza virus, and Newcastle disease virus.

**Table 1:** **CBEP priority EDPs**.

Human health	Animal health	Zoonoses
*Yersinia pestis* (plague) *Francisella tularensis* (tularemia) Crimean-Congo hemorrhagic fever virus Tick-borne encephalitis virus Smallpox virus Clostridium botulinum	Capripox virus Newcastle disease virus African swine fever virus Classical swine fever virus Foot and mouth disease virus *Burkholderia mallei* (glanders) Rinderpest virus Peste des petits ruminants virus	Avian influenza virus *Brucella* spp. *Bacillus anthracis* (anthrax) *Coxiella burnetii* (Q fever)

Cooperative Biological Engagement Program funds two types of research projects: Cooperative Biological Research (CBR) and Threat Agent Detection and Response Activity Projects (TAPs). CBR projects are multi-year endeavors led by non-AJ collaborators with input and support from AJ scientists. To date, two CBR projects have been completed in AJ. The first CBR project (AJ-2) was a clinical, epidemiologic, and laboratory-based assessment of brucellosis that was conducted in collaboration with researchers from the United States Army Medical Research Institute for Infectious Diseases and Louisiana State University. The second CBR project (AJ-3) was aimed at mapping EDPs and was carried out in collaboration with researchers from the University of Florida.

Threat Agent Detection and Response Activity Projects are 1-year undertakings that may or may not include an outside collaborator and are smaller in scope than CBR projects. They are meant to be pilot projects to generate sufficient preliminary data to justify more extensive research projects. Since 2011, seven TAPs have been funded, two of which are complete (Tables 2 and 3). One of the completed projects focused on avian influenza and Newcastle disease viruses [TAP-9 (3)], while the other focused on viral and rickettsial pathogens in arthropod vectors in northern AJ (TAP-8). Five additional TAPs are in various stages of project implementation.

**Table 2:** **Number and progress of projects submitted by CBEP scientists (2011–2016)**.

AJ government entity	Total	Not Selected	In progress	Funded/ongoing	Complete
Ministry of Health	12	9	0	2	1
State Veterinary Control Service	16	12	2	1	1
Joint MoH/SVCS	3	0	1	2	0
Ministry of Defense	1	0	1	0	0
Total	32	21	4	5	2

Initially, CBEP research in AJ centered on CBR projects led by international collaborators. The local scientists were passively involved in these projects – collaborators handled the bulk of the work, including developing research questions, writing proposals, analyzing results, and developing reports and manuscripts. As a result, AJ scientists were not developing the skills needed to sustain their research activities beyond the period of CBEP engagement. In order to increase the capacity of AJ scientists to conduct independent research, in 2011 BNI proposed that CBEP-funded research going forward should center on projects developed by AJ scientists. DTRA approved this approach, and now CBEP is providing extensive mentorship, training, and support to improve the research skills and capabilities of AJ scientists through the implementation of TAPs.

### Grant Writing Mentorship

The mentorship process begins when an AJ scientist submits a research idea to BNI for consideration. BNI works with the scientist to ensure that the research question is clear, achievable, and falls within the CBEP’s scope. Once submitted to and approved by DTRA for further development, the AJ author develops a draft white paper in AJ, which BNI translates to English and reviews, providing feedback to the author that is aimed at improving the quality of the proposal. This phase is highly iterative; most white papers require several rounds of feedback before they are finalized. Telephone conversations and in-person meetings are held with project stakeholders as needed to clarify questions and discuss information to be included in the document. Once stakeholder consensus on the white paper is achieved, the paper is submitted to DTRA for approval; once approved, the draft–review–revise process described earlier is repeated as the AJ author develops a full proposal for DTRA’s consideration. The author prepares a list of materials and supplies to include in the project budget, and a draft sampling schedule that accommodates project duration and participant availability.

Before submission to DTRA, all proposals are approved by the internal scientific review committee of the submitting AJ institution(s). This step helps to ensure that AJ science projects comply with all ministry and institutional procedures and requirements.

Defense Threat Reduction Agency reviewer feedback on submitted white papers and abstracts is verbally communicated pending translation of comments and recommended changes from English to Azerbaijani. Once complete, the AJ author receives a copy of the translated comments and BNI helps him or her revise the proposal to address reviewer concerns. Although time-consuming, this approach fully engages authors in the grant development process and provides AJ scientists with a deeper understanding of research proposal planning and development.

Since 2011, 32 project ideas have been submitted for consideration for CBEP funding (Table 2), of which eleven have been selected for white paper submission. Of those eleven, two have been completed: TAP-8 examined the prevalence of viral and rickettsial pathogens in arthropod vectors in northern AJ, and TAP-9 focused on detection of avian influenza and Newcastle disease viruses in the Barda region of AJ (Table 3). Both TAP-8 and TAP-9 were developed and implemented by AJ scientists with support from CBEP. TAP-10, -11, and -13 are all being implemented (Table 3). TAP-12 and -14 are approved, but sampling has yet to begin.

**Table 3:** **Proposal titles of completed, approved, and ongoing TAP projects (2011–2015)**.

Project	Title	Key words	Ministry	Status
TAP-8	Ecological and epidemiological study of viral and rickettsial pathogen prevalence in arthropod vectors in the northern part of Azerbaijan	Arboviruses, Azerbaijan, vector, arthropods, ticks, sandflies, mosquitoes, rickettsia, Q fever, CCHF, TBE, West Nile virus, PCR	MoH	Completed
TAP-9	Biosurveillance of avian influenza and Newcastle disease viruses in the Barda region of Azerbaijan using real time RT-PCR and hemagglutination inhibition	Avian influenza, Newcastle disease, Azerbaijan, environmental surveillance, live bird market, Hemagglutination inhibition, real-time RT PCR	SVCS	Completed
TAP-10	Ecological and epidemiological study of *Yersinia pestis* and *Francisella tularensis* in the northern part of Azerbaijan regions of Gusar and Khachmaz	*Francisella tularensis, Yersinia pestis*, ticks, fleas, Azerbaijan, PCR	MoH	Ongoing
TAP-11	Regional study of the ecology of anthrax foci in Georgia and Azerbaijan	Anthrax, Azerbaijan, Georgia, genotyping, GIS, bacteriology, PCR	SVCS and MoH	Ongoing
TAP-12	Isolation of *Brucella* species from the milk of lactating ruminants in the Goygol Rayon of Azerbaijan	Brucellosis, Azerbaijan, serology, ELISA, bacteriology, PCR	SVCS	Approved
TAP-13	Investigation of mosquito- and tick-borne arboviruses in Southeastern Azerbaijan	Arboviruses, Azerbaijan, vectors, ticks, mosquitoes, PCR, VectorMap	MoH	Ongoing
TAP-14	Detection and study of anthrax in central lowland region of Azerbaijan	Anthrax, burial sites, Azerbaijan, bacteriology, PCR	SVCS and MoH	Approved

Some of the proposals outlined in Table 2 were not related to CBEP pathogens of interest and were therefore not selected by DTRA for further development. In these cases, BNI suggested other more suitable funding sources for these proposals, as applicable. Consequently, four non-EDP proposals were submitted to the Science Development Foundation under the President of the Republic of Azerbaijan for funding consideration. In some cases, the non-EDP projects received support from other organizations; for example, a rabies project was funded by the UK’s Animal Plant Health Agency and described by Zeynalova et al. (4). These organizations provided funding for research, training programs, and workshops. Thus, different organizations provided additional funding for research, training programs, and workshops.

### Research Mentorship

After a proposal is approved by DTRA, extensive mentorship is provided throughout project implementation. At the beginning of each project, a kick-off workshop is held to align expectations and familiarize project participants with the planned research. AJ scientists also develop data collection forms, which are reviewed by BNI for completeness and used to facilitate data capture. A BNI Science Coordinator (a local national staff member) is assigned to each project to guide participants, periodically accompany the team on field expeditions, and visit the laboratory to ensure that sample collection and diagnostic testing are being conducted safely and appropriately.

Each month, the AJ scientists provide updates on all activities and results for inclusion in a monthly research report to DTRA. This process acquaints project participants with the principles of report writing, data analysis, and effective communication on a routine basis, and prepares them to develop more extensive documents in the later phases of research. These monthly reports also help to identify issues that may require further attention from CBEP in a timely fashion. In addition, the monthly reports provide the raw material for comprehensive quarterly reports and final reports, which can ultimately serve as the basis for publications in international journals. For example, TAP-9, which was conducted by the RVL, was described in a publication by Zeynalova et al. (3). In addition, the final data for TAP-8, conducted by RAPS, are currently being analyzed for subsequent publication. Both projects were implemented by AJ participants with CBEP support during proposal development and research execution.

As with other former Soviet countries, scientific research was affected by the dissolution of the USSR that was indicated by a drop in scientific production after 1991. In 2006–2007, AJ, Kazakhstan, and Ukraine were publishing about half or less the number of scientific articles than they had in the early 1980s (http://www.science-metrix.com/) (5). National research funding for government health-care workers is limited, so international granting agencies are essential sources of support for infectious disease research. The CBEP has provided significant research opportunities to AJ scientists interested in investigating especially dangerous pathogens (outlined in Table 1).

Presentations for international conferences and manuscripts for submission to journals are also subjected to the draft–review–revise mentorship process. AJ scientists have presented project overviews and results of CBEP research at several international conferences/events, including the American Society of Tropical Medicine and Hygiene Annual Meeting, the Institute of Experimental and Clinical Veterinary Medicine, the Annual Meeting of the American Society for Virology, the International Research Conference on Brucellosis, and the Workshop on the Biology of Anthrax. The AJ project team members prepared the presentations and posters for these conferences with support from the BNI Science Team, as described earlier. Mentorship for conference attendance, abstract/poster development, and post-conference trip reports contributes to the scientists’ abilities to meaningfully interact with the international scientific community.

One of the most important components of the CBEP is the enhancement of biosafety and security (BS&S) practices. Each research proposal is therefore accompanied by a protocol risk assessment tool. This tool captures the biorisks associated with a particular project and identifies mitigation activities to ensure that appropriate BS&S practices are employed in the field and in the laboratory. The assessment tool includes sections to describe the BS&S capabilities of the involved laboratories and other project facilities; the equipment and biosafety level to be used (and any required protocol/training/procedure enhancements to that biosafety level); biorisk management programs in place; project personnel and their training and experience levels; planned use of PPE; planned methods of decontamination and waste disposal; and pathogens to be investigated and methods of investigation. The principal investigator provides signature approval of the content of the tool before submission, as a mechanism to help reinforce BS&S compliance. Each project designates one individual to make sure that BS&S best practices are used during project implementation; this person works closely with the laboratory Biosafety Officer to reinforce BS&S compliance in the context of risks and mitigations associated with the research project. Research projects also incorporate research-specific BS&S training in advance of the project to reinforce the principles of safe research, and all CBEP-engaged laboratories receive annual BS&S refresher training regardless of involvement in research. Furthermore, the BNI Science Team provides continuous mentorship during research activities to address any BS&S gaps identified over the course of the project. If gaps are identified, additional training, mentoring, and other measures are taken to correct the deficiencies.

### Grant Development and Scientific Writing Skills

In order to improve overall writing skills and grant/research proposal development skills, BNI coordinated a series of workshops conducted by the Civilian Research Development Foundation Global (CRDF). Topics included grant writing skills, scientific writing skills, and development of posters and abstracts for international conferences.

Two individuals from each CBEP-engaged laboratory were selected to participate in these workshops to ensure that each laboratory is equipped to effectively submit a proposal through CBEP or a non-CBEP research funding source. The participant selection process incorporated feedback from AJ Laboratory Directors and the BNI Science Coordinators, who make regular visits to the laboratories and are familiar with laboratory staff. This effort was undertaken to identify the individuals who were most likely to benefit from the training and to be involved in research proposal development in the future. In addition to participants from the CBEP laboratories, scientists from other AJ organizations working with EDPs or collaborating with the CBEP laboratories in AJ were invited to attend the CRDF workshops. Representatives of local funding sources such as the Science Development Foundation under the President of the Republic of Azerbaijan and representatives from local offices of international funding sources were also invited to attend the workshops in order to introduce their programs and provide details on application requirements. The workshop materials were tailored to AJ with information on specific funding sources available to local scientists.

A total of six workshops were delivered by BNI and CRDF between 2011 and 2015 (Table 4). The first two workshops targeted scientists from ministry-level laboratories and institutes. Subsequent trainings were delivered to laboratory scientists from the regional CBEP-engaged laboratories and ASAU, which is located outside of Baku in Ganja. The most recent training, held in December 2015, focused on a small cadre of 12 research specialists selected by the ministries (6 from MoH and 6 from SVCS) to receive focused training on grant proposal development and submission. The expectation is that these 12 individuals will be able to mentor other AJ scientists on grant development and to assist with grant proposal submissions. A significant amount of workshop time was devoted to describe the proposal submission process for the Broad Agency Announcement (BAA) funding mechanism, through which AJ scientists will be able to apply for CBEP research funding once the integrating contractor is no longer in country.

**Table 4:** **Grant writing workshops in Azerbaijan (2011–2015)**.

CRDF workshop topic	Location	Date	Organizations	Number of participants
Scientific writing and conference presentation skills	Baku	December 2012	RVL, SVCS, RAPS, RCH&E	22
Grant Writing	Baku	September 2013	RVL, SVCS, RAPS, RCH&E, AVSRI	35
Grant writing for regional CBEP laboratories representatives	Ganja	July 2014	ZVLs, APDs, ASAU	20
Advanced workshop on strategies for success in proposal development	Baku	December 2014	RVL, SVCS, ZVLs, RAPS, APDs, RCH&E, AVSRI, ASAU	43
Scientific writing	Baku	May 2015	RVL, SVCS, ZVLs, RAPS, APDs, RCH&E, AVSRI, ASAU	61
Professional skills training workshop strategies for success in proposal writing, making successful presentations and English language for grant writing	Baku	December 2015	RVL, SVCS, RAPS, Khachmaz APD, AVSRI	12
Strategies for success in proposal writing: Submitting to DTRA’s Fundamental Research Broad Agency Announcement (BAA)	Baku	April 2016	RVL, SVCS, RAPS, Khachmaz APD, AVSRI	12

*RAPS, Republican Anti-Plague Station; RVL, Republican Veterinary Laboratory; SVCS, State Veterinary Control Service; ZVL, Zonal Veterinary Laboratory; APD, Anti-Plague Division; RCH&E, Republican Centers for Hygiene & Epidemiology; ASAU, Azerbaijan State Agrarian University; AVSRI, Azerbaijan Veterinary Scientific Research Institute*.

### Identification of Non-CBEP Funding Sources

Bechtel National, Inc.’s Research Coordinator routinely notifies AJ scientists of non-CBEP proposal funding opportunities in an effort to increase the probability that they will submit additional proposals on topics that may not be eligible for CBEP funding. The priorities and interests of the AJ scientists do not always align with CBEP priorities; this is reflected in the high number of research proposals received that focus on non-CBEP subjects. CBEP encourages scientists to pursue grants from non-CBEP sources to study these areas, since doing so provides valuable experience in proposal development and will open up additional opportunities for funding over time. CBEP offers mentorship to AJ scientists as they develop research questions and proposals, and translates information regarding non-CBEP funding opportunities.

As a result of CBEP funding notifications, AJ scientists independently applied for the “Mobility Grant” from the Science Development Foundation under the President of the Republic of Azerbaijan. During one CRDF workshop, CBEP informed workshop participants about the open calls for application to this foundation. Four proposals were subsequently submitted to the foundation, and were all selected for funding. This accomplishment demonstrates that AJ researchers are capable of developing successful proposals when made aware of funding opportunities.

## Discussion

As a result of increased mentorship and training offered through CBEP, AJ scientists are more involved in and capable of developing research ideas, writing grant proposals, and conducting independent research. Although the iterative translate–review–revise process is time-intensive, it represents an investment in the long-term development of critical skills that will enable AJ scientists to continue to develop competitive grant proposals long after CBEP engagement has ended. Overall, the scientists are invested in the projects and they feel more ownership when they, rather than collaborators, are leading the projects. This increased engagement has manifested in active development of abstracts for conferences, in seeking assistance to trouble shoot laboratory issues in real time, and in thinking ahead to where the data might take a project next. The skills they acquire as they develop and implement research projects will be transferable to future research.

### Addressing CBEP Research Goals

Cooperative Biological Engagement Program research priorities are being addressed in part through the implementation of the research program in AJ. These priorities include developing and enhancing sustainable partner country capabilities to (1) employ biorisk management best practices and principles, (2) conduct a modern and proactive disease surveillance mission, (3) comply with international reporting guidelines, and (4) promote and implement the One Health initiative (6).

Another goal of the research program is to foster a modern and proactive surveillance mission. Activities conducted during research projects, such as sample collection and diagnostic testing, are also relevant to surveillance. EDPs identified in the context of CBEP research projects must be reported via EIDSS. Research projects provide laboratory staff a chance to exercise these surveillance skills on a larger scale than they might otherwise encounter through routine diagnostic activities in their laboratories. In addition, the data collected can be used to establish baseline levels of knowledge on disease prevalence and distribution, and to characterize endemic agents; such information is critical for identifying outbreaks of disease and implementing control and prevention measures.

The last major goal of the program is to promote One Health initiatives. Significantly, some of the CBEP-sponsored research projects are collaborative projects between the MoH and SVCS. Such a novel approach in AJ is beneficial for encouraging communication and cooperation between agencies; during a zoonotic outbreak, ministries may be more likely to work together if they have a history of collaboration.

### Challenges for CBEP Research Activity

Several obstacles to research execution have been identified during the implementation of the CBEP research program. Language barriers represent a significant hurdle. Most AJ CBEP scientists speak little English, which hinders their ability to search and reference international literature, and makes it more difficult to identify non-CBEP funding opportunities. The importance of English language skills has been emphasized during the grant writing workshops. Addressing this need falls out of the scope of CBEP, although the program is actively looking for English language training opportunities to share with the ministries.

Access to international literature is also limited; in part, this derives from not being subscribed to international scientific research journals for reason of cost, lack of access to electronic copies, and so on. This makes it difficult for the scientists who can read English literature to keep up to date on current research methods and techniques. AJ scientists rely heavily on AJ and Russian literature, which is often neither relevant nor comprehensive. The use of online translation software and applications has been incorporated into the grant writing workshops as a means of improving the scientists’ ability to access funding announcements and other information available on the internet.

The competing professional obligations of personnel engaged in research projects present yet another challenge to successful research implementation. BNI has observed that dedicated and engaged scientists tend to be the most interested in research. These same individuals generally have more responsibilities, leaving less time for writing grants and conducting research. This problem exists throughout the system, where the top-performing staff tends to have the bulk of responsibilities. In order to address this challenge, BNI has encouraged regional scientists to take leadership roles in research projects and has encouraged all scientists participating in the grant writing workshops to submit project ideas.

### Sustainability of Research Activities

The efforts summarized in this review represent the first steps in an ongoing process that will ultimately provide AJ scientists with the skills to plan and implement research projects of local and regional relevance. Having the skills and knowledge to develop research plans and apply for grants independently will allow AJ scientists to receive more funding for research, training, and other programs that might improve national capabilities. The long-term success of the research program will be highly dependent on Government of Azerbaijan involvement. Finally, the sustainability of research activity will depend on the ability of the MoH and SVCS to maintain laboratory equipment and infrastructure for conducting the proposed research. Research will need to be prioritized, with time allocated for scientists to work on proposal development, grant writing, and execution of research activities. Publications in peer-reviewed journal articles will increase the visibility of the AJ research program within the international scientific community, assist in developing collaborations, and increase the likelihood that scientists will receive funding in the future. In order to sustain research efforts in AJ, local investigators must be able to identify and successfully compete for international funding.

To increase the number of Azerbaijanis trained in grant writing, the final workshop event in early 2016 included a training-of-trainers component. Those who excelled in previous workshops and/or research projects, and who demonstrated an interest in training others were selected to attend this workshop; in all, 12 scientists participated in this phase of training, which began with a workshop conducted in December 2015. It is expected that these trainees will ultimately train other staff at their facilities and develop projects in collaboration with their colleagues.

Bechtel National, Inc. will continue to provide support and mentorship in project development and funding through the end of their period of performance. CBEP is transitioning to an online research proposal submission system using the US BAA process, which will provide a mechanism for AJ scientists to continue to apply for funding after BNI is no longer in the country. BNI mentored scientists on the new process by helping them set up accounts for each of the ministries; the next stage of the process will be to assist with submission of research projects through this new online portal. In addition, BNI’s Research Coordinator will continue to share information on local and international funding sources with the CBEP scientists to encourage engagement outside of DTRA’s purview. By working collaboratively, the challenges to develop a sustainable research program may be overcome for the mutual benefit of all of the participants engaged in this process.

## Author Contributions

All authors listed have made substantial, direct, and intellectual contribution to the work and approved it for publication.

## Conflict of Interest Statement

The authors declare that this work was conducted in the absence of any commercial or financial relationships that could be construed as a potential conflict of interest.
